# Depression contributing to dyslipidemic cardiovascular risk in the metabolic syndrome

**DOI:** 10.1007/s40618-016-0601-y

**Published:** 2016-12-23

**Authors:** A. V. Lemche, O. S. Chaban, E. Lemche

**Affiliations:** 1 Institute of Clinical Research, Berlin, Germany; 2 Section of Psychosomatic Medicine, Bogomolets National Medical University, Kiev, Ukraine; 3 Section of Cognitive Neuropsychiatry, Institute of Psychiatry, Psychology and Neuroscience, King’s College London, Box PO 69, De Crespigny Park, London, SE5 8AF UK

**Keywords:** Metabolic syndrome, Triglycerides, Biomarkers, Zung Self-Rating Depression Scale, Structural equation modeling, National sample, Cross-sectional design, Cohort studies, SCORE

## Abstract

**Purpose:**

Triglycerides are considered an emerging risk factor for cardiovascular mortality. Recent evidence relating depression and metabolic syndrome (MetS) implicated triglyceride levels. We thus investigated interrelations of self-reported depression severity (Zung) and MetS-related biological measures with CVD risk estimates in MetS patients.

**Methods:**

*N* = 101 patients fulfilling International Diabetes Federation criteria for MetS from a nationwide sampled treatment cohort for MetS with familial T2DM risk or manifest T2DM in a Ukrainian governmental health care system were participants. Both laboratory and non-laboratory measures were included. Recent European cardiological SCORE system CVD risk estimates were used as outcome variables.

**Results:**

Following correlation matrix, we entered all variables into principal component analysis (PCA; 76.7% explained variance), followed by hierarchical regression and structural equation modeling (SEM). The PCA suggested a one-factor solution, where the latent variable showed highest loadings of SCORE risk estimates, triglycerides, depression severity, and pulse pressure. A comprehensive SEM was adjusted with 92.7% explained variance: overall CVD risk related to depression, pulse pressure, triglycerides, and fasting glucose.

**Conclusion:**

The findings in this MetS sample suggest that triglycerides and depression severity are the key variables among MetS biomarkers in cross-sectionally associating with the fatal and total SCORE risk estimates in MetS.

**Electronic supplementary material:**

The online version of this article (doi:10.1007/s40618-016-0601-y) contains supplementary material, which is available to authorized users.

## Introduction

There is accumulating evidence for a reciprocal relation between depression and elevated triglycerides (TG) level [1, 2], in general, and specifically in the context of the metabolic syndrome (MetS). Serum TG involvement in MetS is herein a key factor, with (a) free fatty acids promoting insulin post-receptor signaling conducive to insulin resistance, (b) hepatic overproduction of very low-density lipoprotein (VLDL, please refer to list of abbreviations for acronyms) and chylomicron residuals, and (c) formation of small-dense LDL and HDL particles with high atherogenic potential from hepatic triglycerides [3, 4]. MetS is a multicomponent condition where endocrinologic, nutritional, and circulation factors, dependent on genomic-environmental interaction and epigenetic programming, enter with increasing age into a mutually degrading spiral, resulting in insulin resistance, cardiovascular adverse events, and ultimately also in neurodegeneration.

Stress-related hypothalamic-pituitary adrenocortical (HPA) axis over-excitability in MetS contributing to cortisol action on metabolic factors has been documented independently by several research groups [5, 6]. Accordingly, available meta-analyses indicate a notion of bi-directionality [7] between depression and MetS, with longitudinal research providing both support for baseline-depression bearing risk for MetS [1, 8], and support for baseline-MetS bearing risk for depression [2, 9, 10]. Whether plasma lipid traits confer risk to cardiovascular events at all is not uncontroversial, yet have influential prospective cohort studies suggested a role of triglycerides in ischemic cardiovascular diseases (CVD) [11, 12].

Genomic and epigenetic studies also point to interactions among those respective genomic bases, which are in greater probability for expression and translation in the presence of adverse contexts in lifestyle, environmental, and personality circumstances [13]. These are, according to current results, then more likely to form epigenetic templates for factors critical to formation of MetS. For example, there is a robust relation between recurrent depression and obesity [14], with the consequence of dyslipidemia, type 2 diabetes mellitus (T2DM), and CVD. For example, subsequent genomic investigations [15] substantiated an epigenetic alteration of *FTO* gene (16q12.2) polymorphisms, which regulate fat mass and obesity, in the context of depression recurrence. There is also a substantiated epigenetic feedback loop from adiposity to *APOA5* and expression of other genes in locus 11q23 then influencing levels of plasma lipid traits TG, HDL, and LDL [16].

In the present study, we investigated within an observational national Ukrainian MetS cohort the cross-sectional relations of MetS biomarkers with self-reported depression levels and CVD risk probabilities as assessed with the SCORE system of the European Cardiological Society. To estimate respective variance proportions and causal interrelation, structural equation modeling (SEM) was employed. Regarding hypotheses, we generally followed the largely replicated observation that depression is a risk factor attenuating survival following cardiovascular events. As a specific hypothesis, we envisaged a role of serum triglycerides in combination with MetS components and depression toward estimation of cardiovascular risk.

## Materials and methods

### Participants

**Fig. 1:**
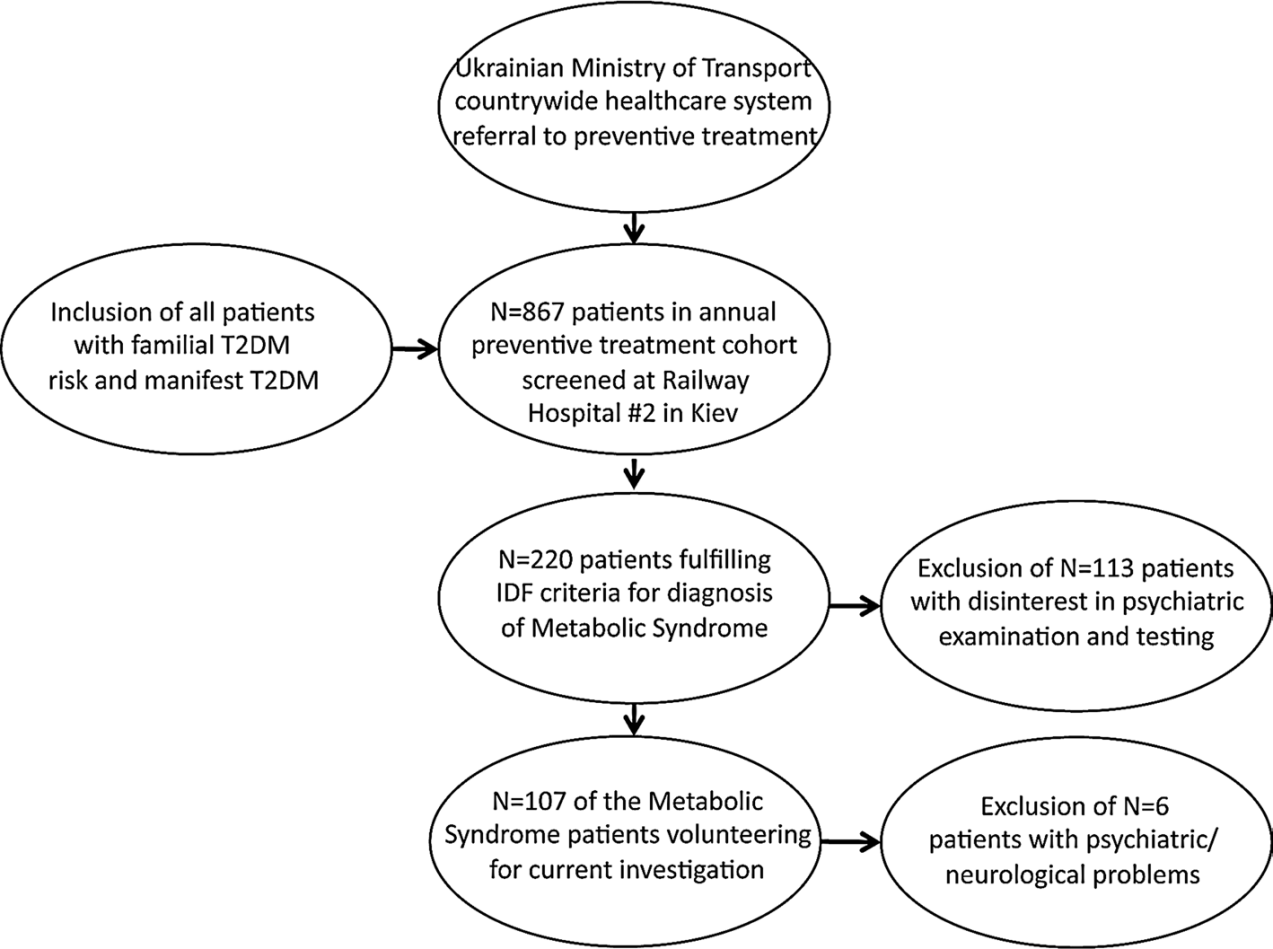
Flowchart for patient sampling and exclusion steps. *Note* The final sample in statistical analyses was *N* = 101

Volunteering participants were recruited from a national pool of MetS patients monitored and treated at specialized units at Railway Hospital No. 2 in Kiev following referral within the countrywide healthcare system of the Ukrainian Ministry of Transport from all parts of Ukraine. Based on endocrinologic parameters or family histories (e.g., parents or siblings affected) indicating specific elevated risk for T2DM, these patients were annually re-examined and treated. Compilation of the final sample *N* = 101 (mean age 45.6 ± 0.14 SEM years; education levels 4.73 ± 0.11, 4 = junior college level, 67 females) is summarized by the flowchart in Fig. 1 illustrating steps with inclusion and exclusion criteria.

### Sample compilation

Composition and size of the final sample was adapted conforming to designs (e.g., [17]) in biomarker detection studies in MetS, specifically in counterbalancing diabetic and non-diabetic patients. The Institutional Review Board of the National Medical University of the Ukraine had endorsed all procedures. All subjects gave written informed consent to the scientific use of their data and were reimbursed for their participation. The investigation was conducted in compliance with the Helsinki Declaration (www.wma.net/e/ethicsunit/helsinki.htm).

### Disease classification

Obesity, hyperinsulinemia, dyslipidemia, and elevated arterial blood pressure, according to ICD-10 criteria, were the primary consensus diagnoses independently established by at least two staff cardiological and/or endocrinologic specialists. International Diabetes Federation (IDF; www.idf.org/publications) MetS criteria were coded from the patient files: Indication of MetS was concluded in the presence of three or more of the following features: waist circumference >80 cm in women and >94 cm in men; fasting serum triglycerides ≥1.7 mmol/L, serum high-density lipoprotein <1.29 mmol/L in women and <1.03 mmol/L in men; systolic blood pressure ≥130/85 mm/Hg or treated; fasting plasma glucose level ≥5.6 mmol/L or T2DM.

### Self-report

The validated official Russian language version of the Zung Self-Rating Depression Scale [18] (ZSDS; www.dic.academic.ru/dic.nsf/ruwiki/1682956) was used for depression self-report. The ZSDS was designed to assess clinical depression with 20-item positively or negatively worded questions addressing somatic, affective, and cognitive aspects of depression on a four-level Likert-type scale. Scores <35 are considered non-depressed; 35–50 mildly, 50–59 moderately, and >60 severely depressed. Other clinical scales were administered for control purposes as previously reported [19, 20]. We focus in this report on results specifically pertaining to depression levels.

### Biological laboratory data

Sitting arterial blood pressure was determined brachially by digital oscillometric sphygmomanometer device upon hospital admission conforming to clinical standard procedures. Pulse pressure was computed as the difference span between SBP and DBP (∆BP). Waist circumference was determined by band measure 1 cm above umbilicus level. Laboratory analyses of all biological specimens were performed in-house with enzymatic methods using commercially available reagents. The laboratory of Railway Hospital No. 2 was subject to quality control standards (e.g., round robin tests) imposed by the Ukrainian Ministry of Public Health and was certified accordingly.

### Establishment of cardiovascular risk variables

Cardiovascular risk estimation was performed based on 2011–2012 criteria of the European Cardiological Society (http://www.escardio.org/guidelines) [21]. The variables individual CVD risk, SCORE fatal risk, and SCORE total risk were established following the instructions. The SCORE system was used with Ukraine classified as a CVD high-risk country. Likewise, following instructions, the variable individual CVD risk was computed by statistical aggregation of seven components. Risk levels for total cholesterol, HDL, LDL, VLDL, Friedewald fraction, triglycerides, etc., were based on 2002 National Institute of Health (NCEP III) criteria and 2013 American College of Cardiology/American Heart Association cholesterol guidelines [22–24] (S1 Supporting Text for more details).

### Statistical methods and models

Using the recommended standard textbook approach [25], we examined the data in the steps correlation, PCA, HRA, and SEM. The SEM module in STATA, which is based on the Bentler–Weeks regression approach [26, 27] to decomposing variance–covariance matrices, employs the maximum likelihood statistics in fitting observed information matrices (OIM) with the Newton–Raphson stepping algorithm. Our overall SEM approach was the specification of a measurement-based causal model, with using the latent construct indicated by the one-factor solution for overall CVD risk in the preceding PCAs. Error terms were included to estimate measurement error for both observed and latent variables. Following detection of skewness in all three outcome risk variables, these were treated for GLM analyses by log-10 transformation in SPSS (see S1 Supporting Text and S5&6 Supporting Tables 2&3).

STATA^®^/MP 12.1 (64-bit version; StataCorp LP, College Station, TX, USA) was used for the statistical methods hierarchical regression analysis (HRA), multivariable logistic regression (MVLR), and structural equation modeling (SEM). IBM SPSS 20 for Intel Macintosh computers (IBM Corp, Armonk, NY, USA) was used to compute correlations, analyses of variance (ANOVA), and principal components analysis (PCA).

## Results

### Scale reliability and confounds of the depression measure

The internal consistency for the ZSDS, as ascertained by Cronbach’s coefficient *α* = 0.824, was satisfactory for further analyses and well in line with previously reported psychometric properties for this instrument. Examination for confounds of ZSDS revealed that family size (i.e., number of persons actually cohabitating, but not the number of children) was significantly associated with greater depression (*r* = 0.252). Females (*M* = 46.36 ± 1.028 SEM) had significantly severer depression (*t* = −2.738, *df* 80.42, *p* = 0.008, 95% CI 7.323–1.158) than males (*M* = 42.12 ± 1.159 SEM). Significant relations with depression severity were also found for height (*r* = 0.201) and waist girth (*r* = 0.347). All other sociodemographic and anthropometric measurements were unrelated (*r*s < 0.2) to depression severity. The range of ZSDS distribution depression severity of this sample (S6 Supporting Figure 2) with a prevalence of ZSDS scores >35 but remaining <60 indicates that the majority in this sample suffers from depression in a mild to moderate strength. Higher depression ranges are missing in this sample in accordance with exclusion criteria (such higher ranges would have indicated severe psychopathology).

### Examination of laboratory and risk variables

Descriptives of all basic study variables are listed (S4 Supporting Table 1). Significant direct associations for biological variables were observed for SBP (*r* = 0.199), pulse pressure (*r* = 0.239), blood pressure risk (*r* = 0.269), trend toward significance were present for Friedewald fraction (*r* = 0.143) and serum triglycerides (*r* = 0.143). Testing for correlation with all other risk variables including microalbuminuria, fasting glucose, total cholesterol, HDL, LDL, and VLDL was insignificant. Of outcome risk variables, zero-order correlations were significant for the latent variable CVD risk (*r* = 0.223), and for computed individual CVD risk. All other risk variables remained insignificant in association with ZSDS, but exhibited strong inter-correlations (*r*s 0.634–0.967, all *p*s < 0.0001). Sociodemographic confounds for biological variables were rare: Family size was a confounder with Friedewald (*r* = 0.215), blood pressure risk (*r* = 0.248), and triglycerides (*r* = 0.217); education level with triglycerides risk (*r* = −0.217); number of children with HDL (*r* = −0.216). Systematic confounds were present for all outcome risk variables individual CVD risk, fatal SCORE, total SCORE, and latent variable CVD risk, with age (*r*s 0.608–0.809, all *p*s < 0.0001), family size (*r*s 0.193–0.350, all *p*s < 0.05), and number of children (*r*s 0.213–0.263, all *p*s < 0.05). All other correlations were insignificant. Group differences with respect to clinically depressed status (S6 Supporting Table 3) and diabetic status (S5 Supporting Table 2) revealed that outcome variables exhibit sensitivity toward the respective clinical status.

### Preparatory analyses

PCA, ANOVA, HRA, and MVLR models were tested in preparation of SEMs, as suggested by standard approaches. The results of these analyses are reported in the S1 Supporting Text file linked to this article. The profile plots of the ANOVAs for sex differences in disease severity of MetS are depicted in S2 Supporting Figure 1. In the resulting one-factor solution was the main latent component 1 dominated by combined CVC risk (S7 Supporting Table 4). Testing the factor loadings systematically by HRA and MVLR models corroborated the findings in the PCA (results reported in S1 Supporting Text).

### Structural equation modeling

**Fig. 2:**
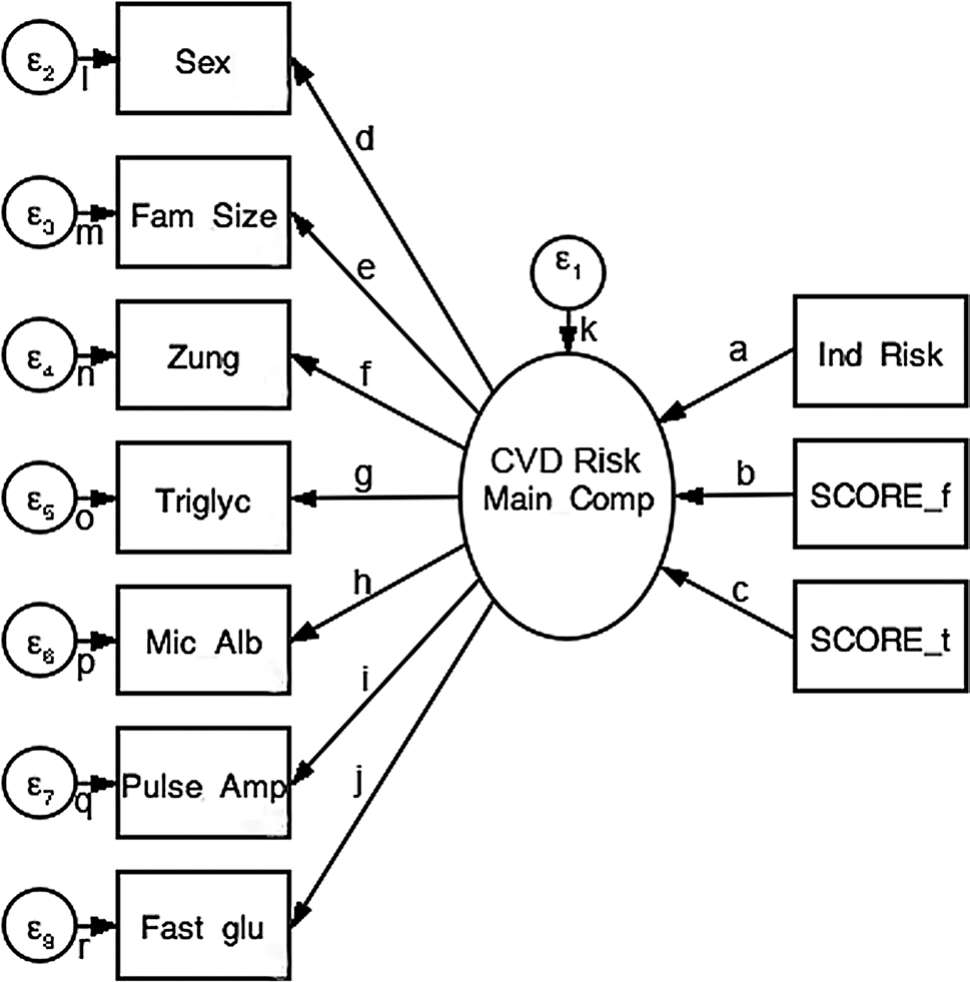
Diagram of comprehensive SEM. *Zung* depressivity score, *Ind Risk* individual CVC risk index, *SCORE_t* total CVD risk index, *SCORE_f* fatal CVD risk index, *ε*_1–8_ error variances, *a*–*r* path indicators (see Table 2)

The aim of the SEM was the setup and testing of a parsimonious causal model with less than ten paths. Steps in SEM included the setup of measurement-based regression path models, and the inclusion of one latent variable (being both exogenous and endogenous), as suggested by the one-factor solution in PCA (i.e., a one-factor causal model). We tested several part models with both relaxed and constrained errors, inclusion of only single outcome risk variables, and with and without adjustment to sex and comorbidities. Although we could obtain valid and stable models, overall percentage of variance explanation remained in ranges *R* ^2^ = 0.461–0.533. The best fitting solution was obtained with a model (a) containing sex as an observed variable, (b) including all three outcome CVD risk variables, (c) including adjustment to comorbidities. This final comprehensive SEM is depicted in Fig. 2, with Table 1 reporting path coefficients and their significances, and Table 2 reporting equation-level goodness-of-fit indices. This constrained model with adjustment of standard errors to differential clustering in comorbidities yielded an overall unique variance explanation of 92.7% in robust OLS path estimation. The adjusted model was distinctly superior to all other solutions (additional information S1 Supporting Text). Please note that none of the traditional SEM goodness-of-fit criteria are reported by STATA in robust estimation. In conclusion, we can state that, out of our computations, the extended and adjusted model yielded best fit, as indicated by the Bentler–Raykov squared multiple correlation coefficients, and the magnitudes in the prediction-correlations (Table 2).

**Table 1:** Comprehensive structural equation model: path estimation

Code	Path	Coefficient	Standard error	*Z*	*P*	95% CI lower	95% CI upper
a	Individual risk → main component	0.141	0.0154565	9.17	0.000	0.111	0.171
b	SCORE fatal → main component	−2.945	0.2305982	−12.77	0.000	−3.397	−2.493
c	SCORE total → main component	3.287	0.1515965	21.69	0.000	2.990	3.584
d	Main component → subject sex	0.453	0.0479932	9.45	0.000	0.359	0.547
e	Main component → family size	0.309	0.0408251	7.59	0.000	0.229	0.389
f	Main component → Zung depression score	0.540	0.0312704	17.28	0.000	0.479	0.601
g	Main component → triglycerides	0.427	0.0326967	13.09	0.000	0.363	0.492
h	Main component → microalbuminuria	0.071	0.0097205	7.29	0.000	0.052	0.089
i	Main component → pulse pressure	0.551	0.0617042	8.94	0.000	0.431	0.672
j	Main component → fasting plasma glucose	0.364	0.0305370	11.92	0.000	0.304	0.423
k	*ε*_1_ error main component	0.073	0.0630226	–	–	0.013	0.395
l	*ε*_2_ error subject sex	0.794	0.0435319	–	–	0.713	0.884
m	*ε*_3_ error family size	0.904	0.0252976	–	–	0.885	0.954
n	*ε*_4_ error Zung depression score	0.707	0.0338024	–	–	0.644	0.777
o	*ε*_5_ error triglycerides	0.816	0.0279834	–	–	0.763	0.873
p	*ε*_6_ error microalbuminuria	0.994	0.0680616	–	–	0.992	0.997
q	*ε*_7_ error pulse pressure	0.695	0.0222248	–	–	0.825	0.912
r	*ε*_8_ error fasting plasma glucose	0.073	0.0630216	–	–	0.013	0.395

^a^Maximum likelihood estimation, 12 iterations; log pseudolikelihood −364.2662

**Table 2:** Equation-level goodness-of-fit indices

Dependents	Fitted	Predicted	Residual	*R* ^2^	mc	mc^2^	Wald-*χ* ^2^	*df*	*P*
Subject sex	0.181	0.372	0.143	0.206	0.453	0.205	–	–	–
Family size	1.342	0.129	1.213	0.095	0.309	0.095	424.09	1	0.0000
Zung depression score	86.991	25.412	61.578	0.293	0.540	0.292	769.79	1	0.0000
Triglycerides	0.675	0.123	0.551	0.183	0.428	0.183	1130.25	1	0.0000
Microalbuminuria	0.195	0.001	0.195	0.005	0.071	0.005	84.85	1	0.0000
Pulse pressure	132.074	40.188	91.936	0.304	0.551	0.304	511.55	1	0.0000
Fasting plasma glucose	4.053	0.536	3.516	0.132	0.363	0.132	1937.60	1	0.0000
Main component	0.037	0.034	0.003	0.926	0.962	0.926	1074.60	3	0.0000
Overall				0.927					

*mc* correlation between the dependent variable and its prediction, *mc* ^2^ the Bentler–Raykov squared multiple correlation coefficient. Stability analysis yields a stability index = 0: SEM satisfies stability condition

*P* value tested against random permutation

## Discussion

The main findings of this study are that, as SEM suggests, overall CVD risk in MetS is associated with an ensemble of single biomarkers (triglycerides, microalbuminuria, fasting glucose level, pulse pressure), single sociodemographic markers and mid-range depression. The results of this study specifically highlight the role of triglycerides in dyslipidemia associated with self-reported depression level as contributing to overall CVD risk in MetS with 29.3% explained variance accounted for. With respect to hypothesis testing, the results in this MetS sample provide further support for manifold findings that depression level is predictive for CVD-related mortality (here indicated by SCORE fatal estimates). In this sample, the pattern of cross-sectional association is such that triglycerides rather than the other lipid traits (cholesterol fractions) exerted the major risk contributing 18.3% to overall CVD risk of 92.7%. As triglycerides are still seen as an “emerging risk factor” (NCEP III guidelines of 2002, ESC guidelines of 2012), this finding provides relative novelty to respective accumulating evidence.

Regarding the relation of depression and mortality, there are consistencies with published literature in this respect. There is an increased risk for mortality (OR 1.5) [28] according to the recent meta-analyses, where CVD is among the main causes of mortality associated with depression. Also, for a relation of depression symptoms and dyslipidemia there is replicated support. A recent Finnish longitudinal developmental study spanning preschool measurement with adulthood indicated that trajectories of steep increases in triglycerides were predictive of depression onset [29]. One prospective study in congenital heart disease for major adverse cardiovascular events (fatal and hospitalization incidences) related to depression indicated ORs 1.6–3.6 for a 5-year period [30]. The cross-sectional fatal SCORE risk in a Finnish regional study for depression was OR 2.9 for males and OR 1.3 for females with 30% of MetS prevalence [31]. In myocardial infarction survivors increases depression onset the longitudinal risk for subsequent cardiovascular adverse events at a HR 1.7, with proportional higher risk for more depressed patients [32]. Also, for stroke, moderate depression assessed with ZSDS resulted in independent risks (ORs 1.9–2.7) [33]. Likewise, in a Dutch 10-year longitudinal study, BDI depression scores rather than actual clinical depression, proved as a better independent predictor, with HR 3.9 for cardiac mortality and 1.7 for non-fatal cardiac events [34].

Also in healthy individuals is depression (ZSDS) associated with inflammation marker C-reactive protein (CRP), leukocytes, and fibrinogen, as a regional Greek study found [35]. In stable CHD patients proved ZSDS a better predictor at OR 1.1 of later adverse cardiovascular events than CRP baseline levels, also in a Greek two-step study [36]. In all studies, depression is found an independent predictor of CVD risk, but MetS components such as diabetes or abdominal obesity also form significant interactions [37]. Psychosocial stress and depression (ZSDS) have associations with different biomarkers, but triglycerides may be a linking variable: in an Italian study, depression correlated with triglycerides, which were also associated with stress scores [38].

The best supported explanatory mechanism for the elevated CHD risk in depression is a dysregulated HPA axis, involving sympathetic overstimulation and hypercortisolemia [39]. HPA dysfunction and MetS criteria were more prevalent in the melancholic subtype of clinical depression [40]. Reduction in telomere length as a result of stress is associated both to single MetS components, including triglycerides, and also progression into MetS constellation [41]. In structural neuroimaging, volume reduction in the nucleus globus pallidus correlated both with MetS severity and depression severity, thus providing a putative brain correlate of MetS [42] (S1 Supporting Text).

One strength of this study lies in countrywide sampling and the high level overall explained variance in SEM. The current study is limited due to its sample size and cross-sectional design, albeit provides robust statistical effects. Further limitations lie in the non-inclusion of direct neuroendocrine measurement and lack of genotype control.

## Conclusion

This study presents evidence in support of the hypothesis that levels of triglycerides play a major role in overall CVD risk in MetS. Manifold evidence has long described depression, CVD-related mortality in the context of MetS symptoms as long-term consequences of dysregulated HPA. The present findings add to these postulates by estimating risks and showing patterns of association cross-sectionally. Future studies should use prospective design and be controlling for genotype and indices of signaling in neuroendocrine pathways in question.

## Electronic supplementary material

Below is the link to the electronic supplementary material.

Supplementary material 1 (DOCX 3968 kb)

## Abbreviations

MetS: Metabolic syndrome
T2DM: Type 2 diabetes mellitus
BMI: Body mass index
CVD: Cardiovascular diseases
CHD: Coronary heart disease
SEM: Structural equation model
PCA: Principal component analysis
HPA: Hypothalamic pituitary adrenocortical axis
ICD: International Classification of Diseases
IDF: International Diabetes Federation
SBP: Systolic blood pressure
DBP: Diastolic blood pressure
∆BP: Difference blood pressure, pulse pressure
GLM: General linear model
HRA: Hierarchical regression analysis
MVLR: Multi-variable logistic regression
ANOVA: Analysis of variance
TG: Triglycerides
HDL: High-density lipoprotein
LDL: Low-density lipoprotein
VLDL: Very low-density lipoprotein
MA: Microalbuminuria
TCH: Total serum cholesterol
OLS: Ordinary least squares
ZSDS: Zung Self-Rating Depression Scale
OR: Odds ratio
HR: Hazard ratio
BDI: Beck Depression Inventory
CRP: C-reactive protein
