# Supplementary material for: Depression contributing to dyslipidemic cardiovascular risk in the metabolic syndrome
Source: J Endocrinol Invest. 2016 Dec 23;40(5):539–46. doi: 10.1007/s40618-016-0601-y (PMC5390000; doi:10.1007/s40618-016-0601-y)
Supplement: Supplementary file 1 — Supplementary material 1 (DOCX 3968 kb) [file 40618_2016_601_MOESM1_ESM.docx]

**S1 Supporting Information**

**Computation and estimation of CVD risk variables**

The risk estimates Individual CVD risk, SCORE fatal risk, and SCORE total risk were computed from the laboratory measurements following the 2011-2012 ECS/EAS guidelines “The Task Force for the management of dyslipidemias of the European Society of Cardiology (ESC) and the European Atherosclerosis Society (EAS)“ (*doi:10.1093/eurheartj/ehr158*). The variable Individual CVD risk, based on the 2004 Lipid-Liga (*www.lipid-liga.de*) criteria, consistent with National Cholesterol Program Adult Treatment Panel III Guidelines*,* was computed from variables subject age, cholesterol quotient, LDL-HDL quotient, triglycerides, microalbuminuria, diabetic status and blood pressure by statistical aggregation (cholesterol quotient ≥4.5, triglycerides ≥2.258, SBP ≥140, DBP ≤95; LDL-HDL quotient was computed using sex-specific cutoff values 3.5 and 4, respectively). To this end, based on cutoff-levels for respective single risk values, risk variables were generated, which were then combined by adding and dividing the single componential values by seven, the number of the single risk variables. The Friedewald fraction and VLDL were computed by using respective standard formulae (e.g. *www.lipid-liga.de*) solely for purposes of statistical comparison. The SCORE estimates for percentages fatal and total, respectively, were derived as described in the SCORE manual using the tabulated estimates. As smoking status was not available in the original data files, the smoking status was simulated by assigning reported national rates in a pseudo-randomized manner, using WHO tobacco control data (*www.euro.who.int/_data/assets/E92554.pdf*).

**Confounding of self-report with disease severity**

To test whether self-reported depression index ZSDS was confounded by disease severities, we set up a series of ANOVAs with illness severity contrasts (burden of complications, number of comorbidities, compensation of diabetes, severity of diabetes, severity of MetS). No significant effects in the depression level are evident for complications, (*F*(8, 101)=0.89, *ns*, *R^2^*=0.078). For *ZSDS* and *comorbidities*, there was a main effect of comorbidities (*F*(6, 101)=2.84, *p*<0.01, *R^2^*=0.153). There was a significant main effect for compensation of diabetes (*F*(3, 101)=2.93, *p*<0.03, *R^2^*=0.083), but not for severity of diabetes, nor for severity of MetS. There was a significant main effect of sex in *ZSDS* (*F*(1, 101)=6.50, *p*<0.01). Given these effects we adjusted for comorbidities and sex in the comprehensive final SEM. **S2 Supporting Figure 1** then depicts profile plots of the two-way ANOVAs by age group and sex differences (Helmert contrasts).

**Ascertainment of latent variables**

Beforehand exploratory factor analyses had revealed that the correlation matrix is decomposable into one factor that exhibited the by far largest eigenvalue. If eigenvalues >1 would be included, five main components would have resulted. However, only the first component had reasonable variance explanation. Therefore, the final PCA was constrained to one factor. The final PCA over all computed CVD risk variables, and including biological risk markers, yielded a main component that accounted for 76.746% of the total variance: **S4** **Supplementary** **Table 3** shows eigenvalues and final factor loadings. In sum, the latent component is dominated by (a) SCORE total, (b) SCORE fatal, (c) pulse pressure, and (d) Zung SDS scores for depression severity: These four variables have eigenvalues >1.

**Exploration of path models by Hierarchical Regression Analysis**

The robust OLS method implemented in STATA was used to re-model factor loadings with multiple regression analyses. In the HRA model for the latent variable, overall CVD risk (*F*=7.36, model-*p*<0.00001, adjusted *R^2^*=0.43) was predicted by ZSDS (*t*=-3.05, term-*p*<0.004, 95%CIs-0.0560--0.0115) age (*t*=4.59, term-*p*<0.0001, 95%CIs0.1909-0.4871), and microalbuminuria (*t*=3.17, term-*p*<0.003, 95%CIs0.2835-1.2580). In the HRA model for SCORE total risk (*F*=755.95, model-*p*<0.00001, adjusted *R^2^*=0.98), SCORE_t was also predicted by ZSDS (*t*=3.73, term-*p*<0.005, 95%CIs0.0025-0.0136), by age (*t*=4.24, term-*p*<0.0001, 95%CIs0.0412-0.1153), by TG (*t*=2.08, term-*p*<0.04, 95%CIs0.0023-0.1274), by pulse pressure (*t*=4.44, term-*p*<0.0001, 95%CIs0.0056-0.0150), and by fasting glucose (*t*=2.94, term-*p*<0.005, 95%CIs0.01240-0.0657). In the HRA for SCORE fatal risk (F= 512.57, model-*p*<0.00001, adjusted *R^2^*=0.98), there were significant terms for ZSDS (*t*=3.48, term-*p*<0.001, 95% CIs0.0041-0.0154), age (*t*=-2.06, term-*p*<0.04, 95%CIs0.0011-0.0763), pulse pressure (*t*=3.87, term-*p*<0.0001, 95%CIs0.0044-0.01389), and fasting glucose (*t*=2.56, term-*p*<0.01, 95%CIs0.0074-0.0616). In the HRA for individual CVD risk (F=413.09, model-*p*<0.00001, adjusted *R^2^*=0.97), predictors were ZSDS (*t*=4.09, term-*p*<0.0001, 95%CIs0.0059-0.01738) and pulse pressure (*t*=3.15, term-*p*<0.003, 95%CIs0.0027-0.0123), and fasting glucose (*t*=2.30, term-*p*<0.02, 95%CIs 0.0039-0.0587). In the HRA for subject age (*F*=103.16, model-*p*<0.00001, adjusted *R^2^*=0.91), significant predictors were family size (*t*=3.22, term-*p*<0.002, 95%CIs 0.2019-0.8693), and pulse pressure (*t*=2.00, term-*p*<0.05, 95%CIs-0.0001-0.0687). In HRA, family size (*F*=99.66, model-*p*<0.00001, adjusted *R^2^*=0.90) was significantly explained by ZSDS (*t*=1.83, term-*p*<0.07, 95%CIs-0.0026--0.0592), age (*t*=3.22, term-*p*<0.002, 95%CIs0.1132-0.4876), and triglycerides (*t*=2.19, term-*p*<0.03, 95%CIs0.0314-0.7128). The HRA for triglycerides (F=144.52, model-*p*<0.00001, adjusted *R^2^*=0.92) revealed family size (*t*=2.19, term-*p*<0.03, 95%CIs0.0185-0.4197), pulse pressure (*t*=2.40, term-*p*<0.02, 95%CIs0.0037-0.0428), and fasting glucose (*t*=2.39, term-*p*<0.02, 95%CIs0.0211-0.2439) as predictors. Likewise, in HRA, pulse pressure (F=267.54, model-*p*<0.00001, adjusted *R^2^*=0.96) was predicted by ZSDS (*t*=4.59, term-*p*<0.0001, 95%CIs0.3557-0.9080), age (*t*=2.00, term-*p*<0.05, 95%CIs-0.0065-4.0182), and by triglycerides (*Z*=2.40, term-*p*<0.02, 95%CIs0.6719-7.5721). In addition, in HRA, fasting glucose levels (F=117.60, model-*p*<0.00001, adjusted *R^2^*=0.92) was predicted by ZSDS (*t*=1.77, term-*p*<0.08, 95%CIs-0.0065-0.1043), triglycerides (*t*=2.39, term-*p*<0.02, 95%CIs0.1149-1.3242), and by microalbuminuria (*Z*=2.51, term-*p*<0.01, 95%CIs0.2957-2.6562). The exact MVLR model for microalbuminuria (4 iterations, *log*-likelihood=-27.7746, *χ^2^*=14.04, *p*<0.02, *R^2^*=0.21) retained as predictors triglycerides (*Z*=1.75, term-*p*<0.08, 95%CIs-0.0953-1.6753), and fasting glucose (*Z*=2.21, term-*p*<0.02, 95%CIs0.0450-0.7469). In sum, it can be stated that regression models confirmed the PCA results by demonstrating reciprocal causal relations amongst the set of biological variables, self-reported depression, and risk estimates.

**Additional SEM information**

When testing the final model using an OIM estimation method, the SEM fit indices were as follows: *χ^2^*=133.391, *df*=39, *p*=0.001, root mean square of estimation RMSEA=0.203 (95%CIs0.165-0.241), Bentler’s comparative fit index CFI=0.433, Tucker-Lewis index TLI=0.389, standardized root mean square of residuals SRMR=0.181, the latter indicating good fit. For a model of this sample size, these fit indices for the adjusted and unconstrained final model are approaching desirable values [1]. Although the *χ^2^* of the relaxed final model was significant, the *χ^2^* divided by the degrees of freedom had a ratio of 3.42, suggesting still a satisfactory fit, which was confirmed by the fact that all other estimates of the model fit parameters obtained values indicating acceptability.

**Additional discussion sections**

New onset depression risk in MetS patients free of CVD is OR 1.7 [2]. However, the baseline prevalence of depression (major depressive episode, current and lifetime) was independently associated with MetS among primary care patients (ORs 1.7~3.7)[3]. In the English Longitudinal Study of Ageing, the ORs 1.5 vs 1.4 for being depressive, was greater for MetS sufferers as compared to metabolically normal obese participants [4]. In a Finnish longitudinal study the OR was 2.2 for new onset depression in MetS sufferers [5]. In humans, MetS is associated with sympathetic and HPA responses. Salivary 3-methoxyphenylglycol, the major metabolite of norepinephrine, has been shown to correlate both with abdominal obesity and depression severity [6].

Experimentation with social suppression in murine models of MetS led to the conclusion that lipogenesis in the liver is significantly upregulated after social defeat, suggesting an interaction of corticosteriods and risky nutrition [7]. In rats, downregulation of hypothalamic insulin receptors induced a metabolic pattern consistent with MetS [8]. The metabolic MetS phenotype exhibited a reduction in neural plasticity, which behaviorally resulted in anhedonia and apathy, similarly to depression. In these animal models, food restriction restituted normal levels of plasma leptin and plasma triglycerides [8]. Experimental manipulation of cortisol uptake in mice produced a metabolic pattern consistent with MetS, namely sudden increases in adiposity, elevated plasma leptin, insulin and triglyceride levels, and sedentary behaviors [9].

**S2 Supporting Figure 1**

Profile plots of ANOVAs for sex differences in disease severities

**S3 Supporting Figure 2**

Distribution of depression scores


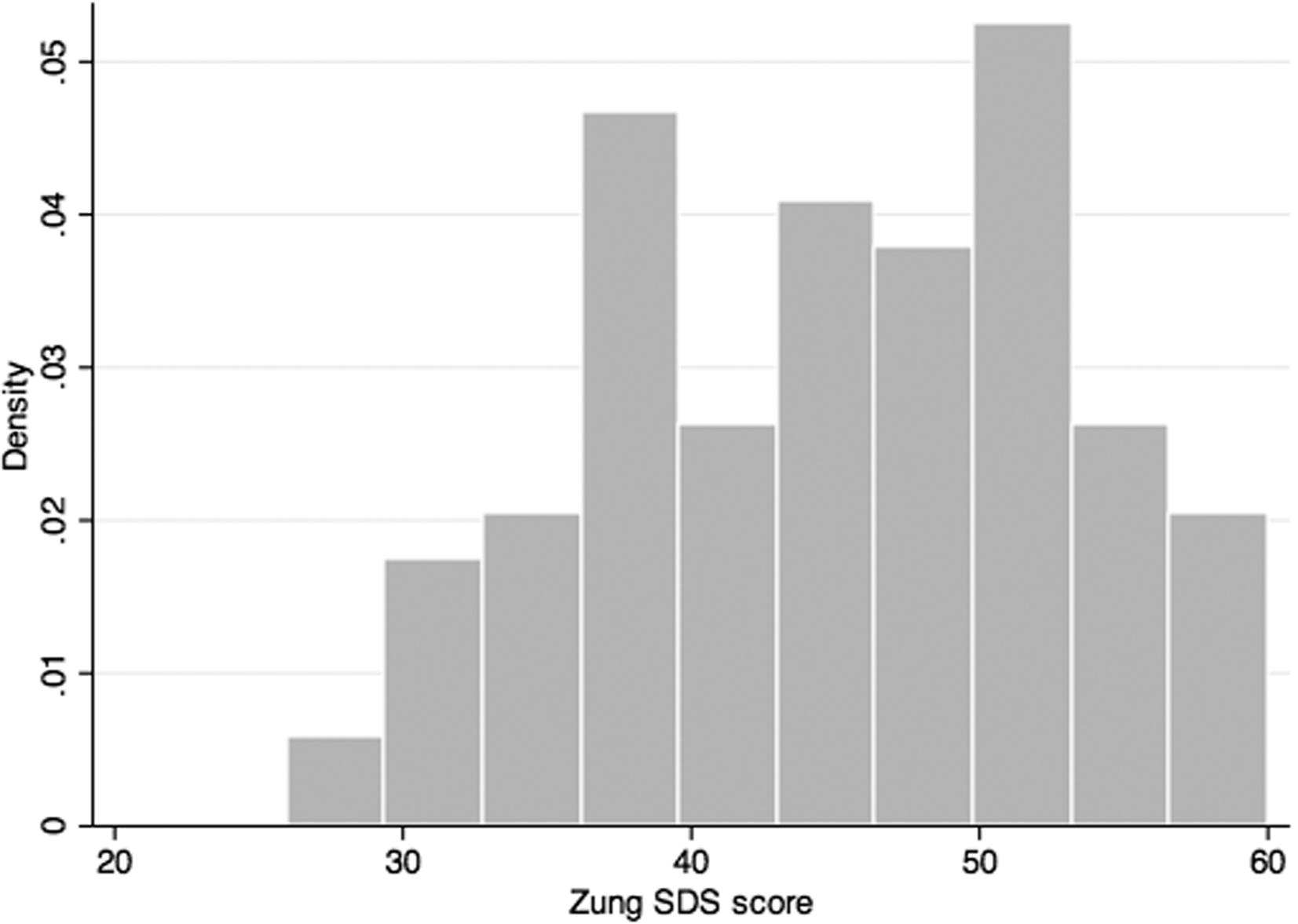


**S4 Supporting Table 1**

Descriptives and intercorrelations of biological measures and risk estimates

| **Variable** | **M±SD** | **Range** | **1** | **2** | **3** | **4** | **5** | **6** | **7** | **8** | **9** |
| --- | --- | --- | --- | --- | --- | --- | --- | --- | --- | --- | --- |
| **1** ZSDS | 44.93±8.113 | 26-60 | 1.0 |  |  |  |  |  |  |  |  |
| **2** ∆BP | 56.633±10.979 | 35-90 | 0.239† | 1.0 |  |  |  |  |  |  |  |
| **3** FMS | 3.20±1.123 | 1-6 | 0.252† | 0.188 | 1.0 |  |  |  |  |  |  |
| **4** FGL | 6.410±1.985 | 4.1-13-3 | 0.078 | 0.097 | -0.015 | 1.0 |  |  |  |  |  |
| **5** MAU | 66.3±0.475 | - | 0.040 | 0.124 | 0.099 | 0.407¶ | 1.0 |  |  |  |  |
| **6** TGL | 3.205±0.855 | 1.6-4.5 | 0.143 | 0.326¶ | 0.217† | 0.327¶ | 0.308‡ | 1.0 |  |  |  |
| **7** ICR | 1.193±0.325 | 0.286-1.857 | 0.218† | 0.425¶ | 0.348¶ | 0.425¶ | 0.453¶ | 0.537¶ | 1.0 |  |  |
| **8** SCF | 10.570±7.194 | 1-47 | 0.017 | 0.476¶ | 0.062 | 0.258¶ | 0.258‡ | 0.305¶ | 0.584¶ | 1.0 |  |
| **9** SCT | 37.940±25.735 | 4-188 | 0.074 | 0.541¶ | 0.111 | 0.330¶ | 0.247† | 0.376¶ | 0.607¶ | 0.969¶ | 1.0 |

Note: ⎯*ZSDS* Zung Self-Rating Depression Scale score; *∆BP* pulse pressure, mm/Hg, *FMS* family size, *FGL* fasting glucose

level, *MAU* microalbuminuria, *TGL* triglycerides, *ICR* Individual CVD risk, *SCF* SCORE fatal risk %, *SCT* SCORE total risk % (raw estimates).

Significance levels †*P*<0.05, ‡*P*<0.01, ¶*P*<0.001 145 words

**S5 Supporting Table 2**

Descriptives and group differences of risk variables between non-diabetic and diabetic patients

| **Variable** | **Non-diabetic M±SD** | **Diabetic M±SD** | ***t*** | ***P*** | **95% CI Lower** | **95% CI Upper** |
| --- | --- | --- | --- | --- | --- | --- |
| Waist circumference | 108.380±23.317 | 97.83±8.208 | 1.185 | ns | -9.5370 | 30.6210 |
| Body Mass Index | 32.775±6.195 | 35.633±4.997 | -0.955 | ns | -9.3869 | 3.6702 |
| Triglycerides | 2.850±1.087 | 3.533±0.937 | -1.260 | ns | -1.8689 | 0.5022 |
| HDL-cholesterol | 0.931±0.141 | 1.011±0.116 | -1.171 | ns | -0.2303 | 0.0695 |
| LDL-cholesterol | 5.9475±1.902 | 6.800±3.578 | -0.530 | ns | -4.6434 | 2.9384 |
| VLDL-cholesterol | 1.1862±0.707 | 1.4761±1.753 | -0.698 | ns | -1.1389 | 0.5586 |
| Friedewald fraction | 0.570±0.217 | 0.706±0.187 | -1.260 | ns | -0.3737 | 0.1004 |
| Systolic blood pressure | 148.75±15.526 | 146.67±8.165 | 0.324 | ns | -12.049 | 16.216 |
| Diastolic blood pressure | 93.13±7.530 | 90.83±7.360 | 0.571 | ns | -6.5380 | 11.121 |
| Pulse pressure amplitude | 55.625±13.741 | 55.833±5.485 | -0.038 | ns | -12.273 | 11.856 |
| Fasting glucose level | 5.825±1.088 | 8.750±2.309 | -2.872 | ns | -5.3575 | -0.4925 |
| Cholesterol quotient | 7.176±1.625 | 6.331±1.697 | 0.939 | ns | -1.1437 | 2.8347 |
| LDL/HDL quotient | 6.308±1.387 | 6.803±3.671 | -0.313 | ns | -1.1438 | 3.3528 |
| Individual CVD risk | 1.035±0.212 | 1.500±0.149 | -6.749 | 0.000 | -0.0103 | -0.0103 |
| SCORE fatal | 12.375±6.501 | 11.500±4.679 | -1.884 | 0.063 | -0.0723 | -0.0019 |
| SCORE total | 40.125±20.138 | 41.000±14.737 | -2.198 | 0.031 | -0.1527 | -0.0077 |
| Main risk component | -0.304±0.622 | 0.542±0.305 | -4.558 | 0.000 | -1.2234 | -0.4801 |

*Note:*—Bootstrapped results based on 995~1000 samples. *Waist* in cm, lipid traits, *fasting glucose* in mmol/L; *blood pressure* measures in

mm/Hg; all risk estimates see Supplement; *main component* is the saved latent regression score derived from principal component analysis.

T-tests in risk variables are based on *log*-transformed variables to ensure normal distributions, unequal variances assumed. 135 words.

**S6 Supporting Table 3**

Descriptives and group differences of biological measures and risk estimates between depressed and non-depressed patients

| **Variable** | **Nondepressed M±SD** | **Depressed M±SD** | ***t*** | ***P*** | **95% CI Lower** | **95% CI Upper** |
| --- | --- | --- | --- | --- | --- | --- |
| TGL | 2.9570±0.8808 | 3.4130±0.7829 | -2.755 | 0.007 | -0.7848 | -0.1276 |
| HDL | 1.0507±0.1173 | 1.0491±0.1231 | 0.065 | *ns* | -0.0462 | 0.0494 |
| LDL | 5.5071±1.5035 | 6.0472±3.0005 | -0.625 | *ns* | -2.3031 | 1.2191 |
| TCH | 6.1630±1.3230 | 6.1580±1.0495 | 0.021 | *ns* | -0.4636 | 0.4733 |
| VLDL | 0.8321±0.4704 | 1.7805±1.9079 | -2.227 | 0.035 | -2.0095 | 0.1129 |
| FWF | 0.5913±0.1761 | 0.6825±0.1565 | -2.755 | 0.007 | -0.1569 | -0.0255 |
| MAU | 0.3000±0.4650 | 0.3600±0.4805 | -0.623 | *ns* | -0.2480 | 0.1300 |
| SBP | 142.83±12.186 | 149.00±13.382 | -2.425 | 0.017 | -11.269 | -1.079 |
| DBP | 89.350±5.7360 | 89.730±5.9640 | -0.324 | *ns* | -2.7030 | 1.9440 |
| ∆BP | 53.478±9.9927 | 59.273±11.156 | -2.752 | 0.007 | -10.013 | -1.5749 |
| FGL | 6.2730±2.1476 | 6.5470±1.8349 | -0.530 | *ns* | -1.3057 | 0.7590 |
| CholQ | 5.9462±1.5463 | 5.9775±0.3934 | -0.107 | *ns* | -0.6121 | 0.5494 |
| LDLHDLQ | 5.3720±1.3001 | 5.8467±2.9422 | -0.567 | *ns* | -1.9369 | 0.9875 |
| CQR | 0.9565±0.2061 | 0.9273±0.2621 | 0.614 | *ns* | -0.0652 | 0.1237 |
| LQR | 0.3043±0.4652 | 0.3455±0.4799 | -0.435 | *ns* | -0.2287 | 0.1463 |
| TGR | 0.6522±0.4815 | 0.8545±0.3558 | -2.425 | 0.017 | -0.3679 | -0.0367 |
| BPR | 0.7609±0.4312 | 0.9273±0.2620 | -2.384 | 0.019 | -0.3048 | -0.0279 |
| ICR | 1.1087±0.3703 | 1.2649±0.2643 | -2.467 | 0.015 | -0.2818 | -0.0305 |
| SCF | 10.111±6.8894 | 10.945±7.4769 | -0.575 | *ns* | -3.7139 | 2.0453 |
| SCT | 34.955±23.281 | 2.9570±0.8808 | -1.067 | *ns* | -15.686 | 4.8340 |
| LVR | -0.288±1.0366 | 0.2363±0.9117 | -2.694 | 0.008 | -0.9120 | -0.1383 |

Note: ⎯*df*=99. *ZSDS* Zung Self-Rating Depression Scale cutoff score=35; *N*_Depressed/Nondepressed_=46/55.

*TGL* triglycerides, *HDL* high-density lipoprotein cholesterol, *LDL* low density lipoprotein cholesterol, *VLDL*

Very low-density lipoprotein cholesterol, *TCH* total cholesterol, *FWF* Friedewald fraction, *MAU* microalbinuria,

*SBP* systolic blood pressure, *DBP* diastolic blood pressure, *∆BP* pulse pressure, *FGL* fasting glucose level,

*CholQ* cholesterol quotient*, LDLHDLQ* LDL-HDL quotient*, CQR* cholesterol quotient risk*, LQR* LDL-HDL quotient risk,

*TGR* triglycerides risk, *BPR* blood pressure risk, *ICR* Individual CVD risk, *SCF* SCORE fatal risk %, *SCT* SCORE total risk %

(raw estimates), *LVR* latent variable CVD risk (regressed factor score). 268 words

.

**S7 Supporting Table 4**

Results of the Principal Component Analysis

| **Rotated Component Matrix^a^** | | |
| --- | --- | --- |
|  | Eigenvalues | Factor loadings |
|  | Component | |
| SCORE total | 20.462  5.663  0.238  0.323  0.142  1.715  6.710  0.631  0.052  0.282 | 0.992  0.948  0.696  0.262  0.307  0.207  0.628  0.316  0.116  0.317 |
| SCORE fatal |  |  |
| Indiv CVD risk |  |  |
| Family size |  |  |
| TG risk |  |  |
| Zung SDS |  |  |
| Pulse pressure |  |  |
| Fasting glucose level |  |  |
| Microalbuminuria |  |  |
| Triglyceride level |  |  |
|  |  |  |

*Note:* ⎯^a^PCA with varimax rotation, one iteration.

Total explained variance 76.746%. KMO=0.650.

61 words

**Supporting References**

1. Bentler PM: **SEM with simplicity and accuracy**. *J Consum Psychol* 2010, **20**(2):215-220.

2. Akbaraly TN, Ancelin ML, Jaussent I, Ritchie C, Barberger-Gateau P, Dufouil C, Kivimaki M, Berr C, Ritchie K: **Metabolic syndrome and onset of depressive symptoms in the elderly: findings from the three-city study**. *Diabetes Care* 2011, **34**(4):904-909.

3. Butnoriene J, Bunevicius A, Norkus A, Bunevicius R: **Depression but not anxiety is associated with metabolic syndrome in primary care based community sample**. *Psychoneuroendocrinology* 2014, **40**:269-276.

4. Hamer M, Batty GD, Kivimaki M: **Risk of future depression in people who are obese but metabolically healthy: the English longitudinal study of ageing**. *Mol Psychiatry* 2012, **17**(9):940-945.

5. Koponen H, Jokelainen J, Keinanen-Kiukaanniemi S, Kumpusalo E, Vanhala M: **Metabolic syndrome predisposes to depressive symptoms: a population-based 7-year follow-up study**. *J Clin Psychiatry* 2008, **69**(2):178-182.

6. Hamer M, Malan L: **Sympathetic nervous activity, depressive symptoms, and metabolic syndrome in black Africans: the sympathetic activity and ambulatory blood pressure in Africans study**. *Stress* 2012, **15**(5):562-568.

7. Chuang JC, Cui H, Mason BL, Mahgoub M, Bookout AL, Yu HG, Perello M, Elmquist JK, Repa JJ, Zigman JM *et al*: **Chronic social defeat stress disrupts regulation of lipid synthesis**. *J Lipid Res* 2010, **51**(6):1344-1353.

8. Grillo CA, Mulder P, Macht VA, Kaigler KF, Wilson SP, Wilson MA, Reagan LP: **Dietary restriction reverses obesity-induced anhedonia**. *Physiol Behav* 2014, **128**:126-132.

9. Karatsoreos IN, Bhagat SM, Bowles NP, Weil ZM, Pfaff DW, McEwen BS: **Endocrine and physiological changes in response to chronic corticosterone: a potential model of the metabolic syndrome in mouse**. *Endocrinology* 2010, **151**(5):2117-2127.

259 words
